# Supplementary material for: Comparative Analysis of Dengue and Zika Outbreaks Reveals Differences by Setting and Virus
Source: PLoS Negl Trop Dis. 2016 Dec 7;10(12):e0005173. doi: 10.1371/journal.pntd.0005173 (PMC5142772; doi:10.1371/journal.pntd.0005173)
Supplement: S1 Text — R code to reproduce the modelling results, including additional models considered as sensitivity analysis. (HTML) [file pntd.0005173.s001.html]

S1 Text: Modelling results


# S1 Text: Modelling results

#### *Comparative analysis of dengue and Zika outbreaks reveals differences by setting and virus*

#### *Sebastian Funk, Adam J. Kucharski, Anton Camacho, Rosalind M. Eggo, Laith Yakob, Lawrence Murray, W. John Edmunds*

- Introduction
  - Required packages
- Generate MCMC chains
- Analyse MCMC chains
- Plots
  - Prepare data
  - Fits
  - Other plots
    - Marginal posterior densities
    - Human-to-human reproduction number vs generation interval
    - Traces
- Other models
  - Reduced population size in Yap
    - Generate MCMC chains
    - Fits
    - Marginal posterior densities
    - Estimated final size for Zika in Yap (proportion infected in the outbreak)
  - Reduced susceptibility against Zika in Yap
    - Generate MCMC chains
    - Fits
    - Marginal posterior densities
    - Estimated final size for Zika in Yap (proportion infected in the outbreak)
  - Two-patch model
    - Fits
    - Marginal posterior densities
    - Fitting the Zika outbreak in Yap in isolation

# Introduction

This document contains the R code necessary to reproduce the results in “Comparative analysis of dengue and Zika outbreaks reveals differences by setting and virus.” All code and data used here are in a github repository. The data sets for the three outbreaks are contained in the `data` subdirectory.

## Required packages

```
cran_packages <- c("cowplot", "dplyr", "tidyr", "stringi", "msm", "rbi")
github_packages <- c("sbfnk/RBi.helpers")
```

```
for (package in cran_packages) {
    install.packages(package)
}

library("devtools")

for (package in github_packages) {
    install_github(package)
}
```

# Generate MCMC chains

Generating MCMC chains requires a working installation of libbi v1.2.0. The model code is in vbd.bi, which is run by the R script dengue\_zika\_mcmc.r.

Edit the lines starting `code_dir <-` and `output_dir <-` in the script ‘dengue\_zika\_mcmc.r’ from the R/ directory in the github repository to point to the correct directories, and run it using

```
Rscript dengue_zika_mcmc.r --nsamples 10000000 --pre_samples 10000 --sample-prior --sample-observations --thin 1000 --fix-natural-history
Rscript dengue_zika_mcmc.r --nsamples 10000000 --pre_samples 10000 --sample-prior --sample-observations --thin 1000 --fix-natural-history --shorter-mosquito-lifespan
```

The first line runs with \(D\_\mathrm{life,M}=2\,\mathrm{weeks}\), and the second one with \(D\_\mathrm{life,M}=1\,\mathrm{week}\)

# Analyse MCMC chains

Set the `code_dir` and `output_dir` to the value used in `sample.posterior.r`

```
output_dir <- "insert your directory here"
code_dir <- "insert your directory here"
```

Load required packages

```
for (package in c(cran_packages, github_packages)) {
    library(tolower(sub("^.*/", "", package)), character.only = TRUE)
}
```

Load script from github repository (add path if necessary)

```
source(paste0(code_dir, "/R/", "analyse_traces.r"))
```

Get MCMC chains of the two libbi runs.

```
libbi_results <- analyse_traces(c("vbd_fnh", "vbd_fnh_shorter"), output_dir)
```

Calculate DIC for models with different mosquito life times (1 week vs. 2 weeks vs. both with equal probability)

```
dic <- sapply(libbi_results, function(x) {
    compute_DIC(x[["trace"]][["posterior"]])
})
dic
```

```
##         vbd_fnh vbd_fnh_shorter     vbd_fnh_all 
##        366.7032        365.8579        366.2954
```

# Plots

## Prepare data

```
ts <- list()
analyses <- data.frame(setting = c("yap", "yap", "fais"), disease = c("dengue", 
    "zika", "dengue"))

for (i in 1:nrow(analyses)) {
    this_setting <- analyses[i, "setting"]
    this_disease <- analyses[i, "disease"]
    this_filename <- paste(code_dir, "data", paste(this_setting, this_disease, 
        "data.rds", sep = "_"), sep = "/")
    this_ts <- readRDS(this_filename) %>% mutate(setting = this_setting, disease = this_disease, 
        week = floor(nr/7))
    ts <- c(ts, list(this_ts))
}

ordered_obs_id_levels <- c("yap_zika", "yap_dengue", "fais_dengue")

data_labels <- ordered_obs_id_levels
data_labels <- sub("^(.*)_(.*)$", "\\2 \\1", data_labels)
data_labels <- sub(" ", " in ", stri_trans_totitle(data_labels))
names(data_labels) <- ordered_obs_id_levels

data <- bind_rows(ts) %>% group_by(week, setting, disease) %>% summarize(value = sum(value), 
    onset_date = min(onset_date)) %>% ungroup() %>% mutate(obs_id = factor(paste(setting, 
    disease, sep = "_"), levels = ordered_obs_id_levels, labels = data_labels)) %>% 
    arrange(week, obs_id) %>% select(week, obs_id, value, onset_date) %>% rename(time = week) %>% 
    mutate(state = "Cases")
first_obs <- data %>% group_by(obs_id) %>% filter(value > 0) %>% slice(which.min(time)) %>% 
    select(time, obs_id) %>% rename(first_obs = time)
last_obs <- data %>% group_by(obs_id) %>% filter(value > 0) %>% slice(which.max(time)) %>% 
    select(time, obs_id) %>% rename(last_obs = time)
data <- data %>% left_join(first_obs, by = "obs_id") %>% left_join(last_obs, 
    by = "obs_id") %>% filter(time >= first_obs & time <= last_obs)
```

## Fits

```
libbi_results[["vbd_fnh_all"]][["trace"]][["posterior"]][["Cases"]] <- libbi_results[["vbd_fnh_all"]][["trace"]][["posterior"]][["Cases"]] %>% 
    mutate(obs_id = factor(obs_id, levels = ordered_obs_id_levels, labels = data_labels))
temp_plot <- plot_libbi(read = libbi_results[["vbd_fnh_all"]][["trace"]][["posterior"]]["Cases"], 
    model = libbi_results[["vbd_fnh_all"]][["model"]], data = data %>% filter(value > 
        0), density_args = list(adjust = 2), extra.aes = list(group = "obs_id"), 
    data.colour = "black", states = "Cases", trend = "mean", plot = FALSE, limit.to.data = TRUE, 
    quantiles = c(0.5, 0.72, 0.95))
obs_states <- temp_plot$data$states %>% inner_join(data %>% select(time, obs_id, 
    onset_date), by = c("time", "obs_id"))
p_obs <- ggplot(obs_states, aes(x = onset_date)) + geom_point(data = data, mapping = aes(y = value)) + 
    facet_wrap(~obs_id, scales = "free") + scale_x_date("Week", labels = scales::date_format("%e %b %Y")) + 
    theme(axis.text.x = element_text(angle = 45, hjust = 1)) + facet_wrap(~obs_id, 
    scales = "free") + scale_y_continuous("Disease incidence") + geom_line(aes(y = value)) + 
    geom_ribbon(aes(ymin = min.1, ymax = max.1), alpha = 0.5) + geom_ribbon(aes(ymin = min.2, 
    ymax = max.2), alpha = 0.25) + geom_ribbon(aes(ymin = min.3, ymax = max.3), 
    alpha = 0.125)

p_obs
```

## Other plots

Define plot labels

```
labels <- c(p_d_inc_h = "italic(D)[plain(inc,H)]", p_d_inc_m = "italic(D)[plain(inc,M)]", 
    p_d_inf_h = "italic(D)[plain(inf,H)]", p_lm = "log[10](italic(m))", p_initial_susceptible_yap = "italic(q)", 
    p_rep = "italic(r)", p_b_h = "italic(b)[H]", p_b_m = "italic(b)[M]", p_t_start = "italic(t[0])", 
    p_pop_yap = "italic(rho)", p_red_foi_yap = "italic(sigma)", p_p_patch_yap = "italic(phi)", 
    R0 = "italic(R)[H %->% H]", GI = "italic(G)", zika = "Zika", yap = "Yap", 
    fais = "Fais")
```

### Marginal posterior densities

```
p <- plot_libbi(read = libbi_results[["vbd_fnh_all"]][["trace"]][["posterior"]], 
    prior = libbi_results[["vbd_fnh_all"]][["trace"]][["prior"]], model = libbi_results[["vbd_fnh_all"]][["model"]], 
    density_args = list(bins = 20, alpha = 0.5, color = "black"), densities = "histogram", 
    extra.aes = list(color = "disease", linetype = "setting"), trend = "median", 
    plot = FALSE, quantiles = c(0.5, 0.95), labels = labels, brewer.palette = "Set1")
p$densities
```

### Human-to-human reproduction number vs generation interval

```
temp_plot <- plot_libbi(read = libbi_results[["vbd_fnh_all"]][["trace"]][["posterior"]], 
    prior = libbi_results[["vbd_fnh_all"]][["trace"]][["prior"]], model = libbi_results[["vbd_fnh_all"]][["model"]], 
    extra.aes = list(color = "disease", linetype = "setting", group = "p_d_life_m"), 
    plot = FALSE, labels = labels, states = c(), params = c("R0", "GI"), noises = c())

r0gi <- temp_plot$data$params %>% filter(distribution == "posterior") %>% mutate(obs_id = tolower(paste(setting, 
    disease, sep = "_"))) %>% filter(obs_id != "fais_zika") %>% mutate(obs_id = factor(obs_id, 
    levels = ordered_obs_id_levels, labels = data_labels)) %>% spread(parameter, 
    value)

cross_sections <- data.frame(GI = c(3, 4))
colnames(cross_sections) <- "italic(G)"

p_r0vgi <- ggplot(r0gi, aes(x = `italic(G)`)) + geom_jitter(aes(y = `italic(R)[H %->% H]`, 
    color = factor(p_d_life_m))) + facet_grid(~obs_id) + scale_x_continuous("Equilibrium generation interval (weeks)") + 
    scale_y_continuous(expression(italic(R)[H %->% H])) + scale_color_brewer("Mosquito life span", 
    palette = "Dark2", labels = c("1 week", "2 weeks")) + theme(legend.position = "top") + 
    facet_wrap(~obs_id, scales = "free") + geom_rect(data = cross_sections, 
    aes(xmin = `italic(G)` - 0.1, xmax = `italic(G)` + 0.1), ymin = -Inf, ymax = Inf, 
    alpha = 0.2, lwd = 3)
p_r0vgi
```

### Traces

```
p$traces
```

# Other models

These are the alternative models considered and mentioned in the manuscript.

## Reduced population size in Yap

### Generate MCMC chains

In this model, the population in Yap is reduced by a factor \(rho\).

Fit the model (remember to update `code_dir` and `data_dir` in `dengue_zika_mcmc.r`):

```
Rscript dengue_zika_mcmc.r --nsamples 10000000 --pre_samples 10000 --sample-prior --sample-observations --thin 1000 --fix-natural-history --sero --pop
Rscript dengue_zika_mcmc.r --nsamples 10000000 --pre_samples 10000 --sample-prior --sample-observations --thin 1000 --fix-natural-history --shorter-mosquito-lifespan --sero --pop
```

Get MCMC chains of the two libbi runs.

```
libbi_results <- analyse_traces(c("vbd_sero_pop_fnh", "vbd_sero_pop_fnh_shorter"), 
    output_dir)
```

### Fits

```
libbi_results[["vbd_sero_pop_fnh_all"]][["trace"]][["posterior"]][["Cases"]] <- libbi_results[["vbd_sero_pop_fnh_all"]][["trace"]][["posterior"]][["Cases"]] %>% 
    mutate(obs_id = factor(obs_id, levels = ordered_obs_id_levels, labels = data_labels))
temp_plot <- plot_libbi(read = libbi_results[["vbd_sero_pop_fnh_all"]][["trace"]][["posterior"]]["Cases"], 
    model = libbi_results[["vbd_sero_pop_fnh_all"]][["model"]], data = data %>% 
        filter(value > 0), density_args = list(adjust = 2), extra.aes = list(group = "obs_id"), 
    data.colour = "black", states = "Cases", trend = "mean", plot = FALSE, limit.to.data = TRUE, 
    quantiles = c(0.5, 0.72, 0.95))
obs_states <- temp_plot$data$states %>% inner_join(data %>% select(time, obs_id, 
    onset_date), by = c("time", "obs_id"))
p_obs <- ggplot(obs_states, aes(x = onset_date)) + geom_point(data = data, mapping = aes(y = value)) + 
    facet_wrap(~obs_id, scales = "free") + scale_x_date("Week", labels = scales::date_format("%e %b %Y")) + 
    theme(axis.text.x = element_text(angle = 45, hjust = 1)) + facet_wrap(~obs_id, 
    scales = "free") + scale_y_continuous("Disease incidence") + geom_line(aes(y = value)) + 
    geom_ribbon(aes(ymin = min.1, ymax = max.1), alpha = 0.5) + geom_ribbon(aes(ymin = min.2, 
    ymax = max.2), alpha = 0.25) + geom_ribbon(aes(ymin = min.3, ymax = max.3), 
    alpha = 0.125)

p_obs
```

### Marginal posterior densities

```
p <- plot_libbi(read = libbi_results[["vbd_sero_pop_fnh_all"]][["trace"]][["posterior"]], 
    prior = libbi_results[["vbd_sero_pop_fnh_all"]][["trace"]][["prior"]], model = libbi_results[["vbd_sero_pop_fnh_all"]][["model"]], 
    density_args = list(bins = 20, alpha = 0.5, color = "black"), densities = "histogram", 
    extra.aes = list(color = "disease", linetype = "setting"), trend = "median", 
    plot = FALSE, quantiles = c(0.5, 0.95), labels = labels, brewer.palette = "Set1")
p$densities
```

### Estimated final size for Zika in Yap (proportion infected in the outbreak)

```
n_infected <- libbi_results[["vbd_sero_pop_fnh_all"]][["trace"]][["posterior"]][["final_size"]] %>% 
    filter(obs_id == "yap_zika") %>% .$value %>% quantile(c(0.025, 0.5, 0.975))
N <- libbi_results[["vbd_sero_pop_fnh_all"]][["trace"]][["posterior"]][["p_N_h"]] %>% 
    filter(setting == "yap") %>% .$value %>% mean
n_infected/N
```

```
##      2.5%       50%     97.5% 
## 0.6820483 0.7246585 0.7699886
```

## Reduced susceptibility against Zika in Yap

### Generate MCMC chains

In this model, the population susceptible to Zika in Yap is reduced by a factor \(r\).

Fit the model (remember to update `code_dir` and `data_dir` in `dengue_zika_mcmc.r`):

```
Rscript dengue_zika_mcmc.r --nsamples 10000000 --pre_samples 10000 --sample-prior --sample-observations --thin 1000 --fix-natural-history --sero 
Rscript dengue_zika_mcmc.r --nsamples 10000000 --pre_samples 10000 --sample-prior --sample-observations --thin 1000 --fix-natural-history --shorter-mosquito-lifespan --sero
```

Get MCMC chains of the two libbi runs

```
libbi_results <- analyse_traces(c("vbd_sero_fnh", "vbd_sero_fnh_shorter"), output_dir)
```

### Fits

```
libbi_results[["vbd_sero_fnh_all"]][["trace"]][["posterior"]][["Cases"]] <- libbi_results[["vbd_sero_fnh_all"]][["trace"]][["posterior"]][["Cases"]] %>% 
    mutate(obs_id = factor(obs_id, levels = ordered_obs_id_levels, labels = data_labels))
temp_plot <- plot_libbi(read = libbi_results[["vbd_sero_fnh_all"]][["trace"]][["posterior"]]["Cases"], 
    model = libbi_results[["vbd_sero_fnh_all"]][["model"]], data = data %>% 
        filter(value > 0), density_args = list(adjust = 2), extra.aes = list(group = "obs_id"), 
    data.colour = "black", states = "Cases", trend = "mean", plot = FALSE, limit.to.data = TRUE, 
    quantiles = c(0.5, 0.72, 0.95))
obs_states <- temp_plot$data$states %>% inner_join(data %>% select(time, obs_id, 
    onset_date), by = c("time", "obs_id"))
p_obs <- ggplot(obs_states, aes(x = onset_date)) + geom_point(data = data, mapping = aes(y = value)) + 
    facet_wrap(~obs_id, scales = "free") + scale_x_date("Week", labels = scales::date_format("%e %b %Y")) + 
    theme(axis.text.x = element_text(angle = 45, hjust = 1)) + facet_wrap(~obs_id, 
    scales = "free") + scale_y_continuous("Disease incidence") + geom_line(aes(y = value)) + 
    geom_ribbon(aes(ymin = min.1, ymax = max.1), alpha = 0.5) + geom_ribbon(aes(ymin = min.2, 
    ymax = max.2), alpha = 0.25) + geom_ribbon(aes(ymin = min.3, ymax = max.3), 
    alpha = 0.125)

p_obs
```

### Marginal posterior densities

```
p <- plot_libbi(read = libbi_results[["vbd_sero_fnh_all"]][["trace"]][["posterior"]], 
    prior = libbi_results[["vbd_sero_fnh_all"]][["trace"]][["prior"]], model = libbi_results[["vbd_sero_fnh_all"]][["model"]], 
    density_args = list(bins = 20, alpha = 0.5, color = "black"), densities = "histogram", 
    extra.aes = list(color = "disease", linetype = "setting"), trend = "median", 
    plot = FALSE, quantiles = c(0.5, 0.95), labels = labels, brewer.palette = "Set1")
p$densities
```

### Estimated final size for Zika in Yap (proportion infected in the outbreak)

```
n_infected <- libbi_results[["vbd_sero_fnh_all"]][["trace"]][["posterior"]][["final_size"]] %>% 
    filter(obs_id == "yap_zika") %>% .$value %>% quantile(c(0.025, 0.5, 0.975))
N <- libbi_results[["vbd_sero_fnh_all"]][["trace"]][["posterior"]][["p_N_h"]] %>% 
    filter(setting == "yap") %>% .$value %>% mean
n_infected/N
```

```
##      2.5%       50%     97.5% 
## 0.6844271 0.7296974 0.7736204
```

## Two-patch model

Fit the model (remember to update `code_dir` and `data_dir` in `dengue_zika_mcmc.r`):

```
Rscript dengue_zika_mcmc.r --nsamples 10000000 --pre_samples 10000 --sample-prior --sample-observations --thin 1000 --fix-natural-history --patch
Rscript dengue_zika_mcmc.r --nsamples 10000000 --pre_samples 10000 --sample-prior --sample-observations --thin 1000 --fix-natural-history --shorter-mosquito-lifespan --patch
```

Get MCMC chains of the two libbi runs.

```
libbi_results <- analyse_traces(c("vbd_patch_fnh", "vbd_patch_fnh_shorter"), 
    output_dir)
```

### Fits

```
libbi_results[["vbd_patch_fnh_all"]][["trace"]][["posterior"]][["Cases"]] <- libbi_results[["vbd_patch_fnh_all"]][["trace"]][["posterior"]][["Cases"]] %>% 
    mutate(obs_id = factor(obs_id, levels = ordered_obs_id_levels, labels = data_labels))
temp_plot <- plot_libbi(read = libbi_results[["vbd_patch_fnh_all"]][["trace"]][["posterior"]]["Cases"], 
    model = libbi_results[["vbd_patch_fnh_all"]][["model"]], data = data %>% 
        filter(value > 0), density_args = list(adjust = 2), extra.aes = list(group = "obs_id"), 
    data.colour = "black", states = "Cases", trend = "mean", plot = FALSE, limit.to.data = TRUE, 
    quantiles = c(0.5, 0.72, 0.95))
obs_states <- temp_plot$data$states %>% inner_join(data %>% select(time, obs_id, 
    onset_date), by = c("time", "obs_id"))
p_obs <- ggplot(obs_states, aes(x = onset_date)) + geom_point(data = data, mapping = aes(y = value)) + 
    facet_wrap(~obs_id, scales = "free") + scale_x_date("Week", labels = scales::date_format("%e %b %Y")) + 
    theme(axis.text.x = element_text(angle = 45, hjust = 1)) + facet_wrap(~obs_id, 
    scales = "free") + scale_y_continuous("Disease incidence") + geom_line(aes(y = value)) + 
    geom_ribbon(aes(ymin = min.1, ymax = max.1), alpha = 0.5) + geom_ribbon(aes(ymin = min.2, 
    ymax = max.2), alpha = 0.25) + geom_ribbon(aes(ymin = min.3, ymax = max.3), 
    alpha = 0.125)

p_obs
```

### Marginal posterior densities

```
p <- plot_libbi(read = libbi_results[["vbd_patch_fnh_all"]][["trace"]][["posterior"]], 
    prior = libbi_results[["vbd_patch_fnh_all"]][["trace"]][["prior"]], model = libbi_results[["vbd_patch_fnh_all"]][["model"]], 
    density_args = list(bins = 20, alpha = 0.5, color = "black"), densities = "histogram", 
    extra.aes = list(color = "disease", linetype = "setting"), trend = "median", 
    plot = FALSE, quantiles = c(0.5, 0.95), labels = labels, brewer.palette = "Set1")
p$densities
```

### Fitting the Zika outbreak in Yap in isolation

Fit the model (remember to update `code_dir` and `data_dir` in `dengue_zika_mcmc.r`):

```
Rscript dengue_zika_mcmc.r --nsamples 10000000 --pre_samples 10000 --sample-prior --sample-observations --thin 1000 --fix-natural-history --patch --disease zika --setting yap
Rscript dengue_zika_mcmc.r --nsamples 10000000 --pre_samples 10000 --sample-prior --sample-observations --thin 1000 --fix-natural-history --shorter-mosquito-lifespan --patch --disease zika --setting yap
```

Get MCMC chains of the two libbi runs.

```
libbi_results <- analyse_traces(c("vbd_patch_fnh_yap_zika", "vbd_patch_fnh_shorter_yap_zika"), 
    output_dir)
```

Fit

```
libbi_results[["vbd_patch_fnh_yap_zika_all"]][["trace"]][["posterior"]][["Cases"]] <- libbi_results[["vbd_patch_fnh_yap_zika_all"]][["trace"]][["posterior"]][["Cases"]] %>% 
    mutate(obs_id = factor(obs_id, levels = ordered_obs_id_levels, labels = data_labels))
temp_plot <- plot_libbi(read = libbi_results[["vbd_patch_fnh_yap_zika_all"]][["trace"]][["posterior"]]["Cases"], 
    model = libbi_results[["vbd_patch_fnh_yap_zika_all"]][["model"]], data = data, 
    density_args = list(adjust = 2), extra.aes = list(group = "obs_id"), data.colour = "black", 
    states = "Cases", trend = "mean", plot = FALSE, limit.to.data = TRUE, quantiles = c(0.5, 
        0.72, 0.95))
obs_states <- temp_plot$data$states %>% inner_join(data %>% select(time, obs_id, 
    onset_date), by = c("time", "obs_id"))

p_obs <- ggplot(obs_states, aes(x = onset_date)) + geom_point(data = data %>% 
    filter(obs_id == "Zika in Yap"), mapping = aes(y = value)) + facet_wrap(~obs_id, 
    scales = "free") + scale_x_date("Week", labels = scales::date_format("%e %b %Y")) + 
    theme(axis.text.x = element_text(angle = 45, hjust = 1)) + facet_wrap(~obs_id, 
    scales = "free") + scale_y_continuous("Disease incidence") + geom_line(aes(y = value)) + 
    geom_ribbon(aes(ymin = min.1, ymax = max.1), alpha = 0.5) + geom_ribbon(aes(ymin = min.2, 
    ymax = max.2), alpha = 0.25) + geom_ribbon(aes(ymin = min.3, ymax = max.3), 
    alpha = 0.125)

p_obs
```

Marginal posterior densities

```
p <- plot_libbi(read = libbi_results[["vbd_patch_fnh_yap_zika_all"]][["trace"]][["posterior"]], 
    prior = libbi_results[["vbd_patch_fnh_yap_zika_all"]][["trace"]][["prior"]], 
    model = libbi_results[["vbd_patch_fnh_yap_zika_all"]][["model"]], density_args = list(bins = 20, 
        alpha = 0.5, color = "black"), densities = "histogram", extra.aes = list(color = "disease", 
        linetype = "setting"), trend = "median", plot = FALSE, quantiles = c(0.5, 
        0.95), labels = labels, brewer.palette = "Set1")
p$densities
```

Human-to-human reproduction number vs generation interval

```
temp_plot <- plot_libbi(read = libbi_results[["vbd_patch_fnh_yap_zika_all"]][["trace"]][["posterior"]], 
    prior = libbi_results[["vbd_patch_fnh_yap_zika_all"]][["trace"]][["prior"]], 
    model = libbi_results[["vbd_patch_fnh_yap_zika_all"]][["model"]], extra.aes = list(color = "disease", 
        linetype = "setting", group = "p_d_life_m"), plot = FALSE, labels = labels, 
    states = c(), params = c("R0", "GI"), noises = c())

r0gi <- temp_plot$data$params %>% filter(distribution == "posterior") %>% mutate(obs_id = tolower(paste(setting, 
    disease, sep = "_"))) %>% filter(obs_id != "fais_zika") %>% mutate(obs_id = factor(obs_id, 
    levels = ordered_obs_id_levels, labels = data_labels)) %>% spread(parameter, 
    value)

p_r0vgi <- ggplot(r0gi, aes(x = `italic(G)`)) + geom_jitter(aes(y = `italic(R)[H %->% H]`, 
    color = factor(p_d_life_m))) + facet_grid(~obs_id) + scale_x_continuous("Equilibrium generation interval (weeks)") + 
    scale_y_continuous(expression(italic(R)[H %->% H])) + scale_color_brewer("Mosquito life span", 
    palette = "Dark2", labels = c("1 week", "2 weeks")) + theme(legend.position = "top") + 
    facet_wrap(~obs_id, scales = "free") + geom_rect(data = cross_sections, 
    aes(xmin = `italic(G)` - 0.1, xmax = `italic(G)` + 0.1), ymin = -Inf, ymax = Inf, 
    alpha = 0.2, lwd = 3)
p_r0vgi
```
